# Analysis of multiplex gene expression maps obtained by voxelation

**DOI:** 10.1186/1471-2105-10-S4-S10

**Published:** 2009-04-29

**Authors:** Li An, Hongbo Xie, Mark H Chin, Zoran Obradovic, Desmond J Smith, Vasileios Megalooikonomou

**Affiliations:** 1 Data Engineering Laboratory, Department of Computer and Information Sciences, Temple University, PA, USA; 2 Center for Information Science and Technology, Temple University, PA, USA; 3 Department of Molecular and Medical Pharmacology, David Geffen School of Medicine, UCLA, CA, USA; 4 Department of Human Genetics, David Geffen School of Medicine, UCLA, CA, USA

## Abstract

**Background:**

Gene expression signatures in the mammalian brain hold the key to understanding neural development and neurological disease. Researchers have previously used voxelation in combination with microarrays for acquisition of genome-wide atlases of expression patterns in the mouse brain. On the other hand, some work has been performed on studying gene functions, without taking into account the location information of a gene's expression in a mouse brain. In this paper, we present an approach for identifying the relation between gene expression maps obtained by voxelation and gene functions.

**Results:**

To analyze the dataset, we chose typical genes as queries and aimed at discovering similar gene groups. Gene similarity was determined by using the wavelet features extracted from the left and right hemispheres averaged gene expression maps, and by the Euclidean distance between each pair of feature vectors. We also performed a multiple clustering approach on the gene expression maps, combined with hierarchical clustering. Among each group of similar genes and clusters, the gene function similarity was measured by calculating the average gene function distances in the gene ontology structure.

By applying our methodology to find similar genes to certain target genes we were able to improve our understanding of gene expression patterns and gene functions.

By applying the clustering analysis method, we obtained significant clusters, which have both very similar gene expression maps and very similar gene functions respectively to their corresponding gene ontologies. The cellular component ontology resulted in prominent clusters expressed in cortex and corpus callosum. The molecular function ontology gave prominent clusters in cortex, corpus callosum and hypothalamus. The biological process ontology resulted in clusters in cortex, hypothalamus and choroid plexus. Clusters from all three ontologies combined were most prominently expressed in cortex and corpus callosum.

**Conclusion:**

The experimental results confirm the hypothesis that genes with similar gene expression maps might have similar gene functions. The voxelation data takes into account the location information of gene expression level in mouse brain, which is novel in related research. The proposed approach can potentially be used to predict gene functions and provide helpful suggestions to biologists.

## Background

Gene expression signatures in the mammalian brain hold the key to understanding neural development and neurological disease. Important insights into gene networks in unicellular systems have been obtained using high-throughput multiplex gene expression methodologies, including microarrays [1], gene chips [2] and serial analysis of gene expression (SAGE) [3]. However, these powerful techniques have not yet been applied to understanding how the genome constructs the three dimensional (3D) structure of multicellular organisms. Classic approaches for mapping neural gene expression patterns include in situ hybridization (ISH) and analyzing reporter genes in transgenic mice [4-7]. These methods can be employed to obtain series of 2-D gene expression patterns, which are stackable for provision of 3-D images. However, such techniques provide single cell resolution but are labor intensive and costly. Comprehensive analysis of gene expression in the normal brain using these methods represents a large undertaking and additional study of disease models is not practicable.

To complement ISH and transgenic methods, a new approach is developed by combining voxelation with microarrays for acquisition of genome-wide atlases of expression patterns in the brain [8]. Voxelation involves dicing the brain into spatially registered voxels (cubes). Each voxel is then assayed for gene expression levels and images are reconstructed by compiling the expression data back into their original locations. It employs high-throughput analysis of spatially registered voxels (cubes) to produce multiple volumetric maps of gene expression analogous to the images reconstructed in biomedical imaging systems. The analysis has revealed a common network of co-regulated genes, and has allowed identification of putative control regions. Although the voxelation approach does not give single cell resolution, it does allow acquisition of expression images in parallel, greatly simplifying co-registration and cross-analysis of multiple genes. In addition, voxelation is much cheaper and faster than traditional approaches.

Related research work suggests that voxelation is a useful approach for understanding how genome constructs the brain. The voxelation instruments and their iterations represent a valuable approach to the genome scale acquisition of gene expression patterns in human and rodent brain. Gene expression patterns obtained by voxelation show good agreement with the known expression patterns. Other related work was done involving the distinguished images between normal and Parkinson's disease (PD) brain structures [9]. The investigation has revealed a common network of co-regulated genes shared between the normal and PD brain. It has also identified gene vectors and their corresponding images that distinguished between normal and PD brain structures, most pertinently the striatum. It implies that gene expression signatures in the mammalian brain hold the key to understanding neural development and neurological disease. Gene expression patterns obtained by voxelation also show good agreement with known expression patterns [1].

**Figure 1:**
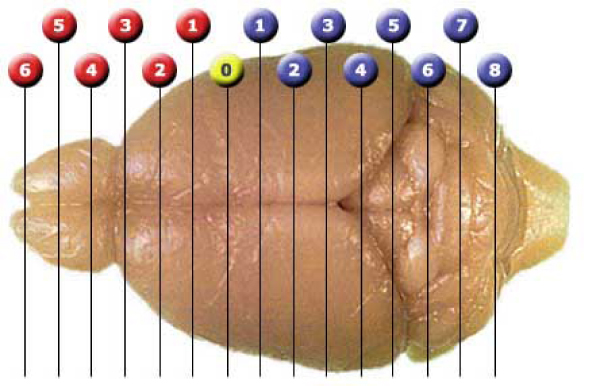
**The mouse brain at bregma = 0**.

Researchers at David Geffen School of Medicine at UCLA used voxelation in combination with microarrays for acquisition of genome-wide atlases of expression patterns in the brain [1,2]. They acquired 2-dimensional images of gene expression for 20,847 genes. The procedure of obtaining the raw data is described here briefly. A freshly sacrificed mouse is taken and removed from its brain. Then a 1 mm thick coronal slice of the mouse brain at the level of the striatum is obtained, which is approximately at bregma = 0 mm and can be visualized in Figure 1.

**Figure 2:**
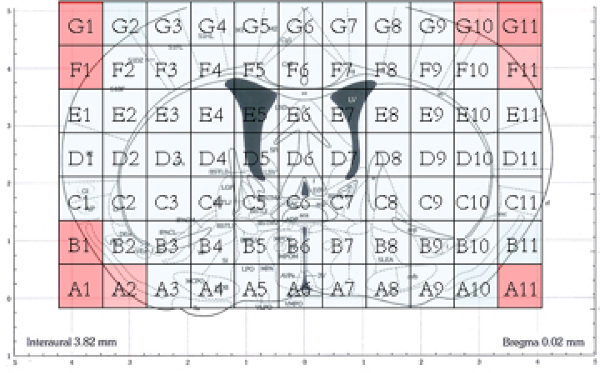
**Voxels of the coronal slice**.

Then the coronal slice is put on a stage and is cut with a matrix of blades that are spaced 1 mm apart thus resulting in cubes (voxels) which are 1 mm3. There are voxels like A3, B9..., as Figure 2 shows. A1, A2... are in red signifying that voxels were not retrieved from these spots, but empty voxels were assigned to maintain a rectangular. So, each gene is represented by the 68 gene expression values composing a gene expression map of mice brain (Figure 2). In other words, the dataset is a 20847 by 68 matrix, in which each row represents a particular gene, and each column is the log2 ratio expression value for the particular probe in a given voxel. The data was found to be of good quality based on multiple independent criteria and insights provided by others into the molecular architecture of the mammalian brain. Known and novel genes were identified with expression patterns localized to defined substructures within the brain.

Previous work [8-10] has been done to detect gene functions, without though taking into account the location information of a gene's expression in a mouse brain to study gene functions. In this paper, we identify the relations between gene expression maps and gene functions based on the 20,847 genes in a coronal slice of the mouse brain. Our analysis consists of similarity queries and clustering analysis of the gene expression maps. The proposed approach is based on the features extracted by the wavelet transform from the original gene expression maps. Among each group of similar genes, we calculate the average gene function distance in the gene ontology structure to indicate the gene function similarity. K-means is used for clustering gene expression maps. The significant clusters that have both similar gene expression maps and similar gene functions are obtained by a proposed technique, which we call multiple clustering. The experimental results from the similarity analysis confirm the hypothesis that genes with similar gene expression map might have similar gene functions. The clustering analysis also detects certain clusters of genes that have similar functions. The proposed approach and analysis can potentially be used to predict gene functions and provide suggestions to biologists.

## Methods

### Basis of methodology

We are investigating the hypothesis that genes with similar expression map have similar gene functions. In order to identify the relationship between maps of gene expression and gene functions, we find genes with similar gene expression maps and check their similarity in gene functions. The methods to estimate the similarity of gene expression maps and the similarity of gene functions are presented below. We also discuss ways to reduce the noise in the raw data set.

### Reducing noise

The original dataset we analyzed consists of data for 20847 genes. Data with no significant gene expression value can be viewed as noise. We eliminate this kind of data to improve the results. If none of the expression values of a gene is bigger than 1 or smaller than -1, we consider the gene insignificant. After normalizing (making sure the mean is 0 and standard deviation is 1) the rest of the data, we obtain a new dataset which has 13576 significant genes. We observe that only half of the genes in the dataset are known genes whose annotation information can be found from an online database, including the function information. The genes with unknown function might confuse our results. So we only consider 7783 genes (from the 13576 significant genes) whose functions are known as the basic dataset for our analysis.

We also take advantage of the inherent bilateral symmetry of the mouse brain by averaging the left and right hemispheres, which proves (as our experimental results demonstrate) very useful in decreasing noise. As Figure 2 shows, we choose the voxels A6, B6, C6, D6, E6, F6 and G6 as midline of the two hemispheres. Then the pairs, (A3, A9), (A4, A8), and (A5, A7) are averaged, and so on. Mice do not have "handedness" or speech-centers of the brain which are known to be localized to one hemisphere in humans. Therefore, a voxel or two that stands out is probably more believable if it has corresponding voxel(s) located in the same general location in the other hemisphere.

### Wavelet features extraction

Each row in the dataset represents the expression values of a given gene for each of the 68 voxels of the particular slice (at the level of the striatum) of mouse brain we consider. A very important step in this analysis is to extract features that characterize each gene expression map. This is done by considering all 68 values in the gene expression map. Intuitively, we expect to have correlation among the values of voxels in the same spatial neighborhood. Moreover, if a voxel's value is similar to other voxels' values in its spatial neighborhood, then we consider it to be more reliable. Working directly with the original 68-element vectors of gene expression values ignores the spatial information. In order to take into account spatial information about the 68 voxels in the brain map, we employ wavelets in feature extraction; wavelets are well known for their properties in conserving local details since they are localized in space.

The wavelet transform is a tool that is used to cut up data, functions or operators into different frequency components and study each component with a resolution matched to its scale [11]. The wavelet transform has advantages over the traditional Fourier transform for representing functions that have discontinuities and sharp peaks; it is also better for lossy compression. The wavelet transform can be classified into discrete wavelet transform (DWT) and continuous wavelet transform (CWT). CWT operates over every possible scale and translation whereas DWT uses a specific subset of scale and translation values. Here, we use the DWT with single-level two-dimensional wavelet decomposition employing the Daubechies D4 wavelet transform to extract features based on the gene expression matrix (Figure 2). The outputs of the wavelet transformation involve approximation coefficients, which are the average of gene expression values in neighborhood voxels, and detail coefficients, which indicate the difference of each voxel from the average. By using multilevel 2-D wavelet decomposition on the 7 by 11 matrix (Figure 2) at level 4, we obtain 75 coefficients including approximation and detail coefficients to achieve the best results.

### Gene maps similarity

Based on the 75 wavelet features extracted from the maps of gene expression, we simply determine the gene maps similarity by calculating the Euclidean distance between each pair of vectors of the 75 features. To rank the different distance values, we define a p-value of this Euclidean distance. Let *S *be a set of Euclidean distances between the query and all the other genes in the dataset, and *Dis *be a special distance between the query and a general gene. Then *Num *is the number of distances *Si*, where *Si *<*Dis*, *Si *∈ *S*. We define the p-value of *Dis *as $\frac{Num}{n}$

where *n *is the number of elements in set *S*. So, for each query, we can find a number of genes which are similar to the query with a corresponding small p-value.

### Gene functions similarity

To identify the functions similarity, we use the average function distance in the gene ontology structure among each group of similar genes. Lin method [16] is used to calculate function distance, i.e. similarity values, between each pair of functions in Gene Ontology structure. The similarity values are obtained within each of the 3 categories of Gene Ontology (GO version: January 2009), and are based on frequencies from the Mouse Genome Informatics (MGI) annotation dataset (MGI version: 01/31/2009). The similarity values are in the range of [0, 1], where 0 denotes that there is no similarity between two functions, and 1 denotes that two gene functions are exactly same.

Because each gene holds more than one gene function, we take all the functions of all the genes in the group to build a set of functions. The average gene function distance is obtained by averaging the distances between each pair of functions in the set; thus, it can be used to determine the function similarity in the group.

For instance, let us consider a group of genes {G1, G2, G3, ....}, where each gene has a number of functions. Assume that G1 has 8 functions {*F1, F2, F3, F4, F5, F6, F7, F8*}, G2 has 7 functions {*F9, F10, F11, F12, F13, F14, F15*}, and G3 has 4 functions {*F16, F17, F18, F19*}, and so on. Then the set of all functions in this group is {*F1, F2, F3, ...., F19, ..., Fn*}, including a total of *n *functions. So the average function distance is defined as:

$$\frac{[\sum_{j=1}^{n}\sum_{i=1}^{n}FunctionSimilarity(Fi,Fj)]-n}{n^{2}-n},$$

where *FunctionSimilarity (x, y) *gives the similarity value between function *x *and function *y*. The similarity value to a function itself is 1 and should be ignored. So we remove n*1 from the sum of similarity values of all function pairs.

To compute p-values of gene function similarity values, we generate a set which is used to rank the function distance values among the randomly selected genes as follows: We randomly choose 1000 gene groups, each consisting of 1000 genes. Then we calculate the average function distance in each group, resulting in a set *U *of 1000 values, called set *rand_func_dis*. For a given average function distance *G_Dis*, the p-value is defined as $\frac{Num\_func}{1000}$,

where *Num_func *is the number of *Ui *with *Ui*<*G_Dis*, *Ui *∈ *U*. So the gene function similarity in a group of genes can be identified by how smaller the p-value of the average function distance of the group is.

### Finding groups of similar genes

We obtain the groups of genes with similar gene expression maps in two different ways. One way is using a typical gene as a query. We choose typical genes as queries and attempt to discover similar genes (w.r.t. the gene expression maps) to the query gene, by using the proposed approach of gene similarity. For each query, we set different values of similarity to get different groups of similar genes. As we mentioned above, the p-value of the Euclidean distance between the wavelet features is used to determine the similarity of gene maps. The smaller p-value we set, the less number of similar genes in the group we found to the query. Then in each group, we check their functional similarity by calculating the average function distance (as defined above).

The other way consists of clustering analysis of the genes and detection of the gene clusters with both similar gene expression maps and similar gene functions. In both of these two ways we need to compute the average function distance for each group of similar genes. Clustering analysis is more complicated and it is described in detail in the following section.

### Multiple clustering

We propose a multiple clustering method to perform the clustering. This method consists of multiple steps. In each step, K-means is used on the current dataset producing *n *clusters. Among the *n *clusters, suppose there are *m *significant clusters (*m*<*n*) whose p-value of average function distance is smaller then 0.05. The new dataset for the next step is obtained by removing the *m *clusters, previously determined as significant, from the current dataset. Then, K-means is repeated again on the newly formed dataset. The process is repeated many times until there are no significant clusters (i.e., with p-value < 0.05) that can be found, or the size of clusters obtained is too small to be meaningful.

### Hierarchical clustering

For the K-means clustering algorithm, the number of clusters is predefined. Without prior knowledge, the estimation of the appropriate number of clusters becomes a challenge in clustering analysis to accurately get the most significant clusters. In this paper divisive hierarchical clustering is used to determine the number of clusters for K-means. In each step of multiple clustering, the number of clusters *n *starts at a minimum value and is incremented. At the first step, *n *starts at 2 and is incremented by 1 until the significant clusters are found. At that time, we assume *n *= K. Then the significant clusters are removed from the dataset and the clustering repeats on the remaining genes. The clustering proceeds to the next step with the number of clusters *n *in this step starting at K-1.

### Clustering analysis

We propose clustering analysis of the gene expression maps and computation of the average function distance in each cluster. Here, we attempt to find the significant clusters that have both similar gene expression maps and similar gene functions. After comparing different clustering methods [12-14], we chose the K-means algorithm [15] as the clustering tool. The proposed clustering method is a combination of multiple clustering and hierarchical clustering (presented earlier).

### Outliers

We consider the genes, which are farthest from the significant clusters as outliers. In order to determine outliers, two conditions are used. One is that the outliers should be farthest from all centers of the significant clusters. The other condition is that the minimum distance between the outlier and all the centers should be maximized too. Since there might be the genes which have biggest sum of distances to all clusters but are very close to one of the clusters, the second condition avoids the situation and restricts the outliers to the genes which are not close to each cluster. To get the outliers, we calculate the distance between all the genes not included in the significant clusters and the average gene map of each cluster. For example, we obtain a set A including the genes which are top 2% (2% is optional) farthest from all the clusters, and get a set B including the genes which have top 2% largest minimum distance from the clusters. The outliers are formed by the intersection of sets A and B.

### Cluster validation

Here we discuss the method that we use to measure the performance of clustering. The point-to-centroid distance is used to determine whether the clusters are compact. The intra-cluster distance is defined as

$$Intra\_Cluster\_Dist=\frac{1}{N}\sum_{i=1}^{k}\sum_{x_{j}\in S_{i}}{\left|x_{j}-\mu_{i}\right|}^{2}$$

where *N *is the total number of data points, *S*_*i*_, *i *= 1,2,.., k, are the *k *clusters and *μ*_*i *_is the centroid or mean point of all the points *x*_*j *_∈ *S*_*i*_.

Another measure of cluster performance is the inter-cluster distance, i.e., the distance between clusters. This is calculated by taking the minimum of the distances between each pair of cluster centroids as follows:

$$\begin{array}{c} \begin{array}{cc} Inter\_Cluster\_Dist=\min\left({\left|\mu_{i}-\mu_{j}\right|}^{2}\right), & i=1,2,...,k-1 \end{array}\\ j=i+1,...,k \end{array}$$

We take the minimum of the distance between clusters because it is the upper limit of cluster performance and is expected to be maximized. The ratio of intra-cluster distance to inter-cluster distance can serve as an evaluation function for cluster performance. Thus, the validity of a *k*-clustering result is defined as

$$Validity=\frac{Inter\_Cluster\_Dist}{Intra\_Cluster\_Dist}$$

Since we want to maximize the inter-cluster distance and minimize the intra-cluster distance, we want the validity value to be maximized.

## Results

### Finding similar genes

**Figure 3:**
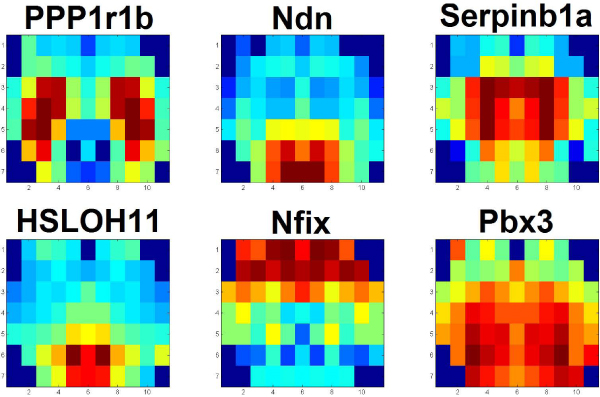
**Typical genes used as queries**.

We selected prototype genes as queries (similarly to [1]), which represent strong but diverse expression patterns and identified genes with similar patterns. Figure 3 shows the gene expression maps and names of the six queries. The six genes are selected [1] as having restricted expression patterns based on the micro-array voxelation data. PPP1r1b is strongly expressed in striatum, Ndn is expressed in hypothalamus, serpinb1a is expressed in striatum, HSLOH11 is expressed in hypothalamus, Nfix is expressed in a gradient pattern in cortex and Pbx3 is expressed in striatum and adjacent ventral structures. Different colors represent different levels of gene expression. Here, we try to find similar genes to a query gene based on the reduced dataset (7783 genes) and the wavelet features.

We consider increasing thresholds of the p-value (from 0.001 to 0.01) and find a number of similar genes whose distance to the target gene is smaller than the threshold. Then, we calculate the average function distance in the group of the selected similar genes. Tables 1, 2, 3, 4, 5, 6 show the results of the six queries. We highlight p-values of function distance that are smaller than 0.05. We consider the function distance with respect to three categories: cellular component, molecular function and biological process.

**Table 1:** Results for Gene PPP1r1b

**P-value of Euclidean Distance**	**Number of similar genes**	**Average Function Distance**					
		
		Cellular Component		Molecular Function		Biological Process	
		Distance	P-value	Distance	P-value	Distance	P-value
**0.001**	7	0.05725	1	0.302341	1	0.349592	0.18
**0.002**	15	0.312983	0.825	0.39233	0.142	0.347346	0.231
**0.003**	23	0.317635	0.714	0.397806	0.061	0.367206	**0.008**
**0.004**	31	0.289464	0.998	0.401542	**0.027**	0.341231	0.385
**0.005**	39	0.320666	0.632	0.403891	**0.016**	0.321738	0.91
**0.006**	47	0.325837	0.491	0.4073	**0.01**	0.307035	0.998
**0.007**	55	0.326283	0.477	0.41908	**0**	0.3157	0.972
**0.008**	63	0.316647	0.747	0.425066	**0**	0.313371	0.986
**0.009**	70	0.315286	0.784	0.406755	**0.01**	0.319979	0.93
**0.01**	78	0.301085	0.977	0.395678	0.087	0.326124	0.822

**Table 2:** Results for Gene Ndn

**P-value of Euclidean Distance**	**Number of similar genes**	**Average Function Distance**					
		
		Cellular Component		Molecular Function		Biological Process	
		Distance	P-value	Distance	P-value	Distance	P-value
**0.001**	7	1	**0**	0.531003	**0**	0.801425	**0**
**0.002**	15	0.272451	1	0.389198	0.222	0.400792	**0**
**0.003**	23	0.290353	0.998	0.377263	0.678	0.396427	**0**
**0.004**	31	0.316816	0.738	0.37295	0.821	0.372537	**0.001**
**0.005**	39	0.305169	0.956	0.381683	0.499	0.37145	**0.002**
**0.006**	47	0.301702	0.975	0.35643	0.996	0.388464	**0**
**0.007**	55	0.317566	0.716	0.362553	0.98	0.363263	**0.021**
**0.008**	63	0.318514	0.692	0.347139	1	0.348138	0.21
**0.009**	70	0.298041	0.988	0.349888	1	0.344336	0.298
**0.01**	78	0.292059	0.997	0.351506	1	0.340159	0.41

**Table 3:** Results for Gene Serpinb1a

**P-value of Euclidean Distance**	**Number of similar genes**	**Average Function Distance**					
		
		Cellular Component		Molecular Function		Biological Process	
		Distance	P-value	Distance	P-value	Distance	P-value
**0.001**	7	0.385133	**0**	0.446529	**0**	0.333031	0.644
**0.002**	15	0.298556	0.987	0.382726	0.46	0.264642	1
**0.003**	23	0.290549	0.998	0.412319	**0.002**	0.285808	1
**0.004**	31	0.309268	0.895	0.41415	**0.002**	0.310873	0.99
**0.005**	39	0.319765	0.658	0.406314	**0.012**	0.299567	1
**0.006**	47	0.285557	1	0.397885	0.06	0.296617	1
**0.007**	55	0.289343	0.998	0.367973	0.927	0.3013	1
**0.008**	63	0.293149	0.997	0.367276	0.938	0.311367	0.99
**0.009**	70	0.294905	0.996	0.376777	0.696	0.320463	0.924
**0.01**	78	0.286614	0.999	0.366045	0.952	0.325361	0.833

**Table 4:** Results for Gene HSLOH11

**P-value of Euclidean Distance**	**Number of similar genes**	**Average Function Distance**					
		
		Cellular Component		Molecular Function		Biological Process	
		Distance	P-value	Distance	P-value	Distance	P-value
**0.001**	7	1	**0**	0.52731	**0**	0.635405	**0**
**0.002**	15	0.45108	**0**	0.419179	**0**	0.423277	**0**
**0.003**	23	0.280144	1	0.381807	0.494	0.403388	**0**
**0.004**	31	0.279249	1	0.378584	0.63	0.415068	**0**
**0.005**	39	0.299816	0.982	0.342651	1	0.402092	**0**
**0.006**	47	0.319518	0.663	0.356403	0.996	0.396327	**0**
**0.007**	55	0.326381	0.475	0.345487	1	0.393314	**0**
**0.008**	63	0.319147	0.675	0.339923	1	0.372925	**0**
**0.009**	70	0.30643	0.942	0.354777	0.998	0.342455	0.351
**0.01**	78	0.274535	1	0.354598	0.998	0.344737	0.284

**Table 5:** Results for Gene Nfix

**P-value of Euclidean Distance**	**Number of similar genes**	**Average Function Distance**					
		
		Cellular Component		Molecular Function		Biological Process	
		Distance	P-value	Distance	P-value	Distance	P-value
**0.001**	7	0.3715	**0.002**	0.414231	**0.002**	0.386766	**0**
**0.002**	15	0.470091	**0**	0.533396	**0**	0.403483	**0**
**0.003**	23	0.327341	0.45	0.446162	**0**	0.393963	**0**
**0.004**	31	0.334632	0.249	0.416353	**0.001**	0.397399	**0**
**0.005**	39	0.354691	**0.029**	0.42041	**0**	0.42754	**0**
**0.006**	47	0.389836	**0**	0.441244	**0**	0.408984	**0**
**0.007**	55	0.379048	**0.002**	0.422284	**0**	0.38242	**0**
**0.008**	63	0.352243	**0.045**	0.428266	**0**	0.358225	**0.05**
**0.009**	70	0.347323	0.066	0.411947	**0.002**	0.355679	0.079
**0.01**	78	0.332507	0.295	0.404723	**0.015**	0.344446	0.295

**Table 6:** Results for Gene Pbx3

**P-value of Euclidean Distance**	**Number of similar genes**	**Average Function Distance**					
		
		Cellular Component		Molecular Function		Biological Process	
		Distance	P-value	Distance	P-value	Distance	P-value
**0.001**	7	0.227326	1	0.499217	**0**	0.305035	0.999
**0.002**	15	0.256744	1	0.372765	0.831	0.307326	0.997
**0.003**	23	0.278943	1	0.394336	0.106	0.341251	0.385
**0.004**	31	0.287043	0.999	0.408898	**0.009**	0.334522	0.599
**0.005**	39	0.312345	0.838	0.378056	0.648	0.34133	0.381
**0.006**	47	0.309653	0.886	0.406041	**0.012**	0.358313	**0.05**
**0.007**	55	0.285096	1	0.385019	0.363	0.353957	0.099
**0.008**	63	0.286593	0.999	0.379561	0.594	0.339088	0.442
**0.009**	70	0.280209	1	0.398416	0.053	0.344987	0.28
**0.01**	78	0.290952	0.998	0.390636	0.176	0.348223	0.209

### Finding significant clusters

**Figure 4:**
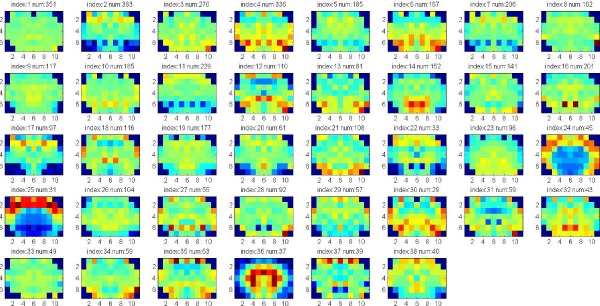
**38 significant clusters found in Cellular Component**.

**Figure 5:**
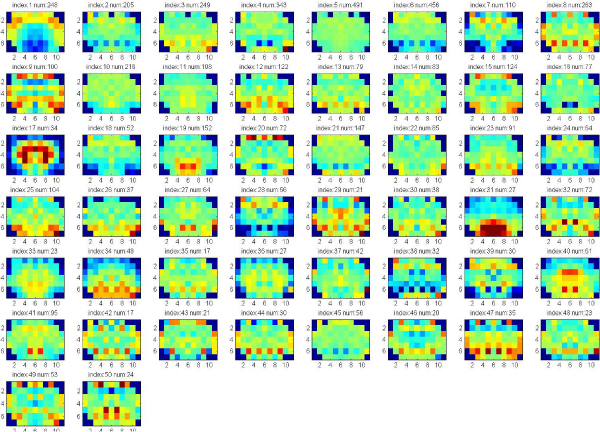
**50 significant clusters found in Molecular Function**.

**Figure 6:**
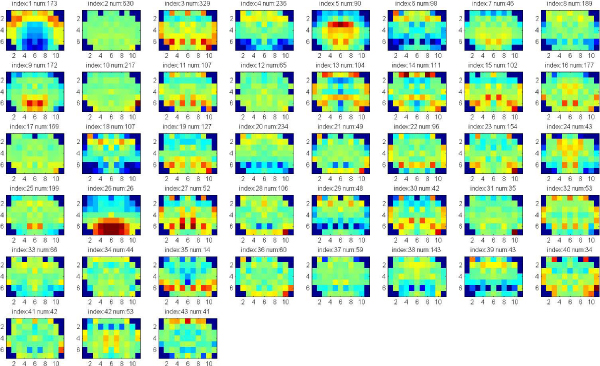
**43 significant clusters found Biological Process**.

In these experiments, we apply clustering iteratively to get the significant clusters with both low p-value (<0.05) of Euclidean Distance of gene expression and low p-value of Function Distance. The experiments are applied on the data set of 7883 genes that consists of both significant and known genes. Each gene is represented by the full 75 wavelet features extracted from the hemi-averaged gene expression map. The multiple clustering combined with hierarchical clustering is repeatedly applied until there are no significant clusters found, or the size of clusters obtained is too small. Figure 4, 5, 6, 7 show the average of gene expression maps of significant clusters obtained by k-means for different ontologies. Each gene expression map corresponds to one cluster.

**Figure 7:**
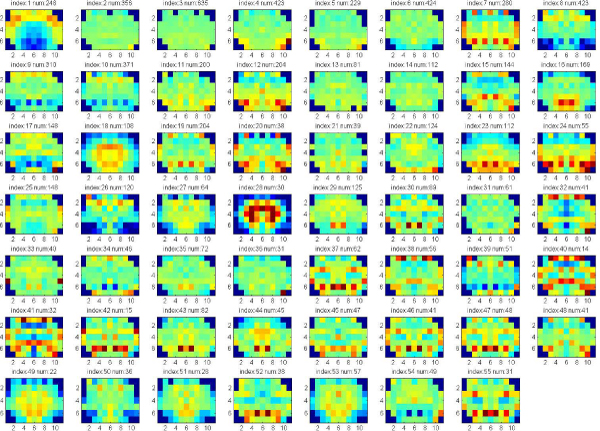
**55 significant clusters found in all the three ontologies**. The three ontologies are Cellular Component, Molecular Function and Biological Process.

Since there are three categories of gene functions in gene ontology, we attempted to identify significant clusters for each one of the three different ontologies (separately) and then with respect to all of the three categories together. For example, when considering the category "Cellular Component", we only searched for significant clusters with low p-value of Functions Distance in the category "Cellular Component". In the case where we considered all three categories together, we searched for significant clusters with low p-value of Functions Distance in any one of the three categories.

### Checking outliers

**Figure 8:**
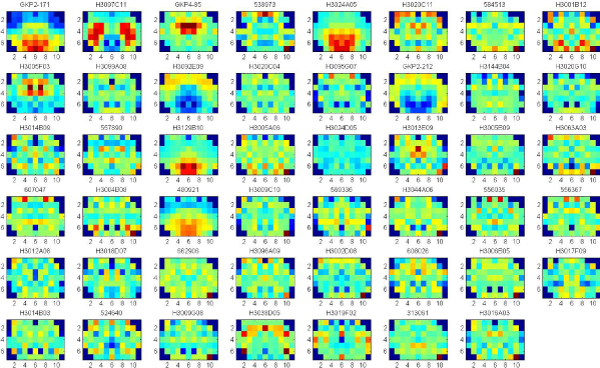
**47 outliers (top2%) of the significant clusters in Cellular Component**. Each gene is identified by Gene Bank ID. The outliers are sorted by the distance from all the significant clusters. The gene names of the top 8 outliers which are located on the first line of the figure (from the left to right) are listed as follows: Neuronatin transcript variant 1, Homo sapiens (PPP1R1B, FLJ20940 fis), Transthyretin, ESTs of Bone marrow macrophage, Mus musculus secreted acidic cysteine rich glycoprotein, Homo sapiens (FLJ13180 fis), ESTs of Transcribed locus, Homo sapiens ring finger protein 2 (RNF2).

**Figure 9:**
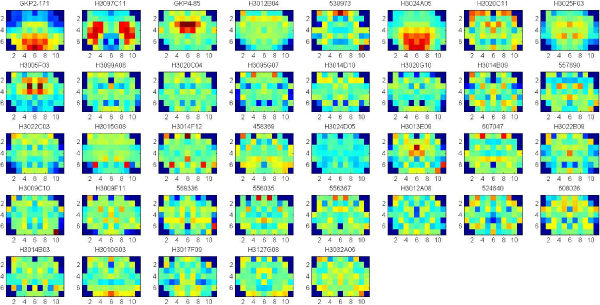
**37 outliers (top2%) of the significant clusters in Molecular Function**. Each gene is identified by Gene Bank ID. The outliers are sorted by the distance from all the significant clusters. The gene names of the top 8 outliers which are located on the first line of the figure (from the left to right) are listed as follows: Neuronatin transcript variant 1, Homo sapiens (PPP1R1B, FLJ20940 fis), Transthyretin, Homo sapiens citrate synthase (CS), ESTs of Bone marrow macrophage, Mus musculus secreted acidic cysteine rich glycoprotein (Sparc), Homo sapiens (FLJ13180 fis), Mus musculus carbonic anhydrase 14 (Car14).

**Figure 10:**
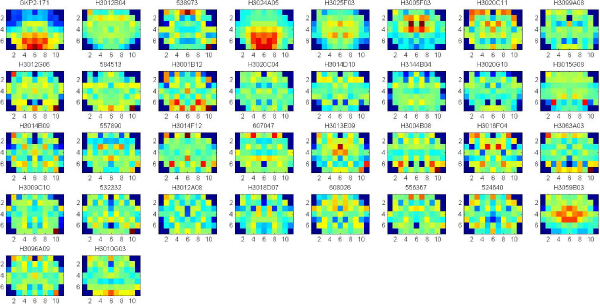
**34 outliers (top2%) of the significant clusters in Biological Process**. Each gene is identified by Gene Bank ID. The outliers are sorted by the distance from all the significant clusters. The gene names of the top 8 outliers which are located on the first line of the figure (from the left to right) are listed as follows: Neuronatin transcript variant 1, Homo sapiens citrate synthase (CS), ESTs of Bone marrow macrophage, Mus musculus secreted acidic cysteine rich glycoprotein (Sparc), Mus musculus carbonic anhydrase 14 (Car14), Mus musculus transthyretin (Ttr), Homo sapiens (FLJ13180 fis), Mus musculus estrogen receptor 1 (Esr1).

**Figure 11:**
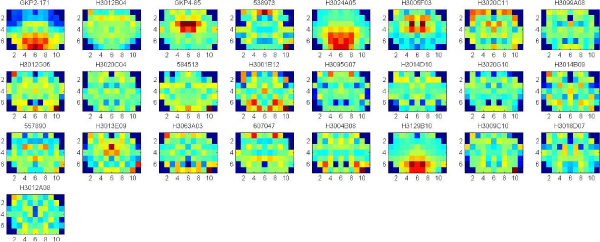
**25 outliers (top5%) of the significant clusters in all the three ontologies**. The three ontologies are Cellular Component, Molecular Function and Biological Process. Each gene is identified by Gene Bank ID. The outliers are sorted by the distance from all the significant clusters. The gene names of the top 8 outliers which are located on the first line of the figure (from the left to right) are listed as follows: Neuronatin transcript variant 1, Homo sapiens citrate synthase (CS), Transthyretin, ESTs of Bone marrow macrophage, Mus musculus secreted acidic cysteine rich glycoprotein (Sparc), Mus musculus transthyretin (Ttr), Homo sapiens (FLJ13180 fis), Mus musculus estrogen receptor 1 (Esr1).

By using the outlier definition (presented earlier), we obtained outliers to the significant clusters, for all the three ontologies. Top 2% outliers were selected to three ontologies respectively, and top 5% outliers were selected for the significant clusters obtained by considering all the three ontologies together. The outliers were sorted by the distance from all the significant clusters. Figures 8, 9, 10, 11 show the gene expression maps of the outliers respectively to different ontologies.

### Cluster validation

In order to evaluate the proposed hierarchical clustering approaches, we used two different clustering algorithms in each step of the multiple clustering to find out the significant clusters. One is k-means with a selected k number, where k is the square root of the size of the data set. The other algorithm is using hierarchical clustering to decide the most suitable k. We evaluated the significant clusters we obtained by calculating cluster distance and compared the results of the two kinds of clustering methods. Table 7 shows that the validity value of the hierarchical clustering (used in our experiments) is larger than the validity value of the selected k clustering in each category.

**Table 7:** Comparing two clustering methods. Intra_Cluster_Dist measures the intra distance inside a cluster, Inter_Cluster_Dist measures the distance between clusters, and Validity indicates the overall performance of the clustering.

**Function Category**	**Method**	**Intra Cluster Distance**	**Inter Cluster Distance**	**Validity**
**Cellular Component**	Selected k	4.0212	0.6355	0.1580
	Hierarchical	4.6096	0.8928	0.1937
**Molecular Function**	Selected k	4.0469	0.5148	0.1272
	Hierarchical	5.0396	1.1211	0.2225
**Biological Process**	Selected k	3.8917	0.6472	0.1663
	Hierarchical	4.7262	0.7971	0.1687
**All the three categories **	Selected k	4.0110	0.5543	0.1382
	Hierarchical	4.8385	0.9813	0.2028

## Discussion

Examining the group of similar genes of target1 (PPP1r1b), Table 1 shows that there are very small p-values of function distance in the category of molecular function, meaning that these similar genes have functions that are very close with respect to position in the gene ontology structure (i.e., these similar genes have similar functions in the category of molecular function). The experimental results of the other targets (Tables 2, 3, 4, 5, 6) also show that genes with similar gene expression maps have very close function position in gene ontology structure, at least in one of the three biological categories. Interestingly, the expression of PPP1R1B, serpinb1a and Pbx3 were most similar to genes in the molecular function ontology, the expression of Ndn, HSLOH11 to genes in the biological process ontology and the expression of Nfix to all three ontologies, that is cellular component, molecular function and biological process.

Using our proposed multiple clustering method, we obtained the significant clusters which have both very similar gene expression maps and very similar gene functions respectively to their corresponding gene ontologies. The cellular component ontology (Figure 4) resulted in prominent clusters expressed in cortex (clusters 24, 25) and corpus callosum (cluster 36). The molecular function ontology (Figure 5) gave prominent clusters in cortex (cluster 1), corpus callosum (cluster 17) and hypothalamus (cluster 31). The biological process ontology (Figure 6) resulted in clusters in cortex (cluster 1), hypothalamus (cluster 26) and choroid plexus (cluster 5). Clusters from all three ontologies combined (Figure 7) were most prominently expressed in cortex (cluster 1) and corpus callosum (cluster 28). It is not surprising that the two most persistent expression patterns are in cortex and corpus callosum, since these regions represent the starkest contrast of tissue in the central nervous system, namely between gray and white matter, respectively.

We also sought genes representing outliers from the expression patterns common to a given gene ontology. Examples of outlier genes from the cellular component and molecular function ontologies were expressed in hypothalamus (neuronatin, transcript variant 1), striatum (PPP1R1B, FLJ20940 fis) and choroid plexus (transthyretin). Neuronatin transcript variant 1 and transthyretin were also outliers from the biological process ontology, although PPP1R1B was not. Together our results suggest that different expression patterns and clusters reflect commonalities and distinctions in various domains of gene function. Thus, valuable clues to function can be obtained from brain gene expression patterns.

## Conclusion

Although research work has been done to detect gene functions, not much effort has focused on identifying the relation between gene expression maps in mice brain and related gene functions. By using wavelet features to determine the similarity of gene expression maps, and the function distance in ontology structure to determine the similarity of gene functions, our analysis on voxelation data showed that the group of genes that was identified as similar to a target gene shares very similar gene functions in at least one gene function category. Moreover, clustering analysis detected certain clusters of genes that have both similar gene expression maps and gene functions. So, the obtained results confirm the hypothesis that genes with similar gene expression map might have similar gene functions. This paper tries to quantify this hypothesis presenting a way to evaluate it as well as a set of genes for which the hypothesis holds.

To obtain the significant clusters, we only analyze the genes, which are both significant and have known functions, i.e., genes whose annotation information can be found at online databases, including the function information. The results based on the dataset we considered support the following claim. By examining the known and unknown genes together to find groups of similar genes (which are obtained either by similarity finding or clustering), one might provide helpful suggestions to biologists about unknown genes having similar gene functions to the known genes in the same group. Therefore the proposed approach has the potential to be used in predicting gene functions.

## List of abbreviations used

PD: Parkinson's disease; UCLA: University of California, Los Angeles; DWT: Discrete wavelet transform; CWT: Continuous wavelet transform; GO: Gene Ontology; MGI: Mouse Genome Informatics.

## Competing interests

The authors declare that they have no competing interests.

## Authors' contributions

LA carried out the data analysis, drafted the manuscript and performed the literature research. HX participated in the design of the study and assisted in the gene function similarity calculations. MC was involved in the data acquisition and interpretation of the results. ZO participated in the conception of the study, in the data analysis and assisted in manuscript editing. DJS conceived the voxelation study, was involved in interpretation of the results and manuscript preparation and assisted in refining the data analysis. VM participated in the conception, design and coordination of the study in the data analysis and assisted in manuscript preparation and editing.
